# Causes of death in two rural demographic surveillance sites in Bangladesh, 2004–2010: automated coding of verbal autopsies using InterVA-4

**DOI:** 10.3402/gha.v7.25511

**Published:** 2014-10-29

**Authors:** Nurul Alam, Hafizur R. Chowdhury, Subhash C. Das, Ali Ashraf, P. Kim Streatfield

**Affiliations:** 1 Centre for Population, Urbanization and Climate Change, icddr,b, Dhaka, Bangladesh;; 2 Formerly with Health Information System Knowledge Hub, School of Public Health, University of Queensland, Brisbane, Australia; 3 Centre for Control of Chronic Disease, icddr,b, Dhaka, Bangladesh; 4 Formerly with Centre for Control of Chronic Disease, icddr,b, Dhaka, Bangladesh

**Keywords:** InterVA, verbal autopsy, cause of death, Abhoynagar, Mirsarai, Bangladesh

## Abstract

**Objective:**

Population-based information on causes of death (CoD) by age, sex, and area is critical for countries with limited resources to identify and address key public health issues. This study analysed the demographic surveillance and verbal autopsy (VA) data to estimate age- and sex-specific mortality rates and cause-specific mortality fractions in two well-defined rural populations within the demographic surveillance system in Abhoynagar and Mirsarai subdistricts, located in different climatic zones.

**Design:**

During 2004–2010, the sample demographic surveillance system registered 1,384 deaths in Abhoynagar and 1,847 deaths in Mirsarai. Trained interviewers interviewed the main caretaker of the deceased with standard VA questionnaires to record signs and symptoms of diseases or conditions that led to death and health care experiences before death. The computer-automated InterVA-4 method was used to analyse VAs to determine probable CoD.

**Results:**

Age- and sex-specific death rates revealed a higher neonatal mortality rate in Abhoynagar than Mirsarai, and death rates and sex ratios of male to female death rates were higher in the ages after infancy. Communicable diseases (CDs) accounted for 16.7% of all deaths in Abhoynagar and 21.2% in Mirsarai – the difference was due mostly to more deaths from acute respiratory infections, pneumonia, and tuberculosis in Mirsarai. Non-communicable diseases (NCDs) accounted for 56.2 and 55.3% of deaths in each subdistrict, respectively, with leading causes being stroke (16.5–19.3%), neoplasms (13.2% each), cardiac diseases (8.9–11.6%), chronic obstructive pulmonary diseases (5.1–6.3%), diseases of the digestive system (3.1–4.1%), and diabetes (2.8–3.5%), together accounting for 49.2–51.2% points of the NCD deaths in the two subdistricts. Injury and other external causes accounted for another 7.5–7.7% deaths, with self-harm being higher among females in Abhoynagar.

**Conclusions:**

The computer-automated coding of VA to determine CoD reconfirmed that NCDs were the leading CoD with some differences between the sites. Incorporating VA into the national sample vital registration system can help policy makers to identify the leading CoDs for public health planning.

Reliable and up-to-date population-based statistics on morbidity and mortality by cause are important for health sector planning to combat ill health and ill-health-induced poverty. Such statistics, to our knowledge, are lacking in Bangladesh, except for children aged under 5 and maternal deaths for which data are available through periodic special health surveys (1–4). More than 70% of the population in Bangladesh lives in rural areas, where most deaths (>88%) occur at home and death certificates from which one can derive causes of death (CoD) are hardly available (5–7). The Health Management Information System of the Ministry of Health and Family Welfare publishes service statistics on morbidity and mortality based on mostly public hospital registry. Deaths with physician-certified cause in public hospitals are a tiny non-representative fraction (<4%) of more than 1 million deaths each year (8). Since 1982, the nationally representative sample vital registration system (SVRS) administered by the Bangladesh Bureau of Statistics records CoD reported by family members of the deceased (7). This non-scientific lay-reporting of CoD, with a high proportion being classified as either unspecified or ill-defined, seriously limits the utility of such information for health sector planning.

In settings such as Bangladesh, where civil registration of deaths is incomplete and medical certification of death is not common practice, verbal autopsy (VA) is a scientific, practical, and low-cost approach to generating population-based information on CoD for health sector planning, implementing, and monitoring (9, 10). Longitudinal demographic surveillance sites, national health surveys, and sample vital registration schemes are increasingly using VA to generate vital statistics with CoD (1–4, 6, 11, 12).

The International Centre for Diarrhoeal Disease Research, Bangladesh (icddr,b) introduced the VA questionnaires in 2004 in its longitudinal sample health and demographic surveillance system (HDSS) maintained in two rural sites in Bangladesh. The sites are located in two different climatic zones. The VA data from these sites provide a rare opportunity to compare the pattern of CoD in two rural populations with varying climates and socioeconomic conditions, but comparable public primary health care facilities and service delivery system.

The objectives of the study are to compare death rates and cause-specific mortality fractions (CSMFs) in males and females between the two rural HDSS sites, as well as the rank-order of CSMFs between age groups. The spatial difference in CSMFs may reveal the importance of such statistics for planning of local public health services.

## Methods

### Location of HDSS sites

**Fig. 1:**
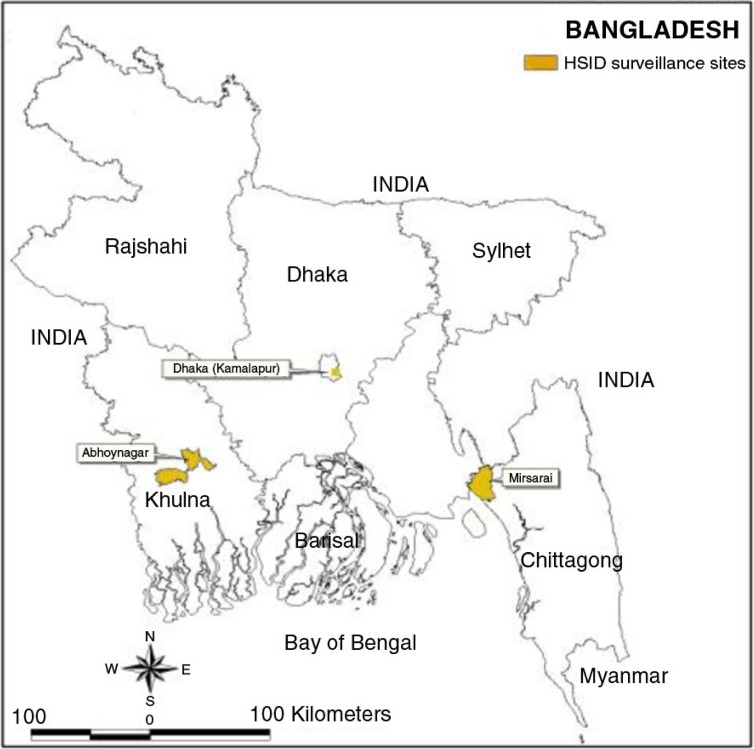
Map of the two health and demographic surveillance sites in Bangladesh.

A member of the INDEPTH Network in Bangladesh, AMK HDSS, has two active rural field sites (Fig. 1). Abhoynagar is 30 km north of Khulna city on the Khulna–Jessore axis where industrialisation and urbanisation are taking place. Mirsarai is 60 km north of Chittagong seaport city on the Dhaka–Chittagong axis, and is on the coast of the Bay of Bengal. Bangladesh is divided into seven distinct climatic zones (13). Abhoynagar is located in the southwestern zone, which is characterised by heavy dewfall; and Mirsarai is in the southeastern zone characterised by more frequent severe hail storms, northwesterly winds and tornadoes, and heavy winter dewfall. Temperatures differ considerably, with the southwestern zone showing average temperatures in January and April of 19°C and 29°C, respectively, and the southeastern zone showing temperatures of 20°C and 28°C in the same months, respectively. Annual rainfall is substantially less in the southwestern zone, 150 cm compared to 250 cm in the southeastern zone. Weather may affect health and CoD differently in different zones.

### Socioeconomic condition

The primary and secondary school enrolment rates of boys and girls in 2008 were comparable between the sites (14). Agriculture is the predominant occupation in each site, with engagement in agriculture being substantially higher in Abhoynagar. Female labour force participation was higher in Mirsarai than Abhoynagar (11% vs. 2%). Households in Mirsarai more frequently reported additional income through remittances than in Abhoynagar (77% vs. 52%). The self-reported average household income and per capita income was higher in Mirsarai compared to Abhoynagar.

### Local health delivery system

The Government of Bangladesh provides primary health care services to all Bangladeshis for a nominal fee through a three-tiered health service delivery system: the Community Clinics, each for about 6,000 people; the Health and Family Welfare Centres, each for 25,000 people; and the Upazila (subdistrict) Health Complexes with an out-patient and an emergency department, 50 in-patient beds, and an operating room, each covering around 250,000 people. Both sites have similar public primary health care infrastructures.

### Sampling in HDSS

The sample HDSS set up by icddr,b in Abhoynagar, covered 122 villages in 7 out of 17 Unions (each has about 25,000 people) since 1982; and in Mirsarai, covered 119 villages in 7 out of 16 Unions since 1994. A stratified, two-staged, random, systematic sampling design is used in each stratum – a subdistrict. The first stage was a random sample of unions and the second stage was a systematic random sample of households (14). A household listing operation was carried out in selected unions to prepare the sampling frame for selection of households. Each household had an equal probability of selection in each stratum. The sample fraction was every sixth household in the Abhoynagar field site and every fourth household in the Mirsarai field site.

### Introduction of VA into HDSS

The standard VA questionnaires developed by the WHO and modified by INDEPTH for neonatal, child, and adult deaths were introduced into AMK HDSS in 2004 to generate population-based vital statistics and CoD of neonates, children, and adults. VA refers to method of interviewing close family members and caretakers of the deceased about the events surrounding the fatal illness episodes or conditions. The interview attempts to unearth what happened during the hours, days, or months preceding the death event. The standard VA contains both open narratives related to death and leading questions to elicit symptoms and signs of illness or conditions of the deceased. For neonatal deaths, a description of the mother's delivery is recorded. These signs and symptoms or conditions alone or in combination are highly indicative of specific disease.

The VA tools in English were customised to suit local conditions and then translated into Bangla. Customisation includes reducing the number of questions on HIV/AIDs and malaria as the prevalence of these two diseases is very low. The VA tools were revised in 2009 to be comparable with the 2008 WHO-revised VA tools. Collection of vital events including VA was approved by the Ethical Review Committee of icddr,b.

### Training of field research assistants

Female field research assistants (FRAs – eight in Abhoynagar, and nine in Mirsarai) having at least higher secondary education were provided intensive training on collection of vital events and migration information. A public-health physician and a medical sociologist provided 4 days’ training to FRAs and field research supervisors (FRSs) of non-medical background on modular VA tools, followed by 2 days of field practice. FRAs with a standard consent form informed the closest caretakers and relatives about the purpose of the study and guaranteed confidentiality of the information they would provide. Willingness to take part was expressed by signature or thumb impression.

### Identification of deaths and VA collection

Each FRA visited about 1,200 households quarterly to record vital events: births, deaths, migrations, and marriages and marital disruptions. During quarterly household visits of FRAs for recording vital events, deaths were identified and VA interviews were conducted. With their consent, FRAs interviewed the closest caretakers and relatives who had lived with the deceased in the same household around terminal illness or death using the VA questionnaire within 6–12 weeks after the date of death. In absence of the main caretaker or relative for a long period, a member of the same *bari* (a group of households close to each other by relationship) was interviewed. FRSs regularly supervised the fieldwork, and the public-health physician was available to provide technical support, such as clarification of questions when required.

### Quality control

Scheduled revisits to 5% of randomly selected households were part of the quality control measures. FRSs visited the FRAs during data collection and reviewed surveillance data collected at household level, including the VA data. Immediate feedback was provided. The research officer completed mandatory checks and edits of all events before sending them for entry to the Surveillance and Data Resources Unit in Dhaka.

### VA data management

HDSS data including VA were entered by three staff using R-base (DOS Version 3.0) software, under the supervision of two data management officers. The software was customised and allowed for inconsistency checks of the data during entry. All inconsistencies detected during data entry were resolved by checking the original forms or by returning the forms with errors to the field sites for necessary checking and corrections. Collected vital events were edited for consistencies and added to the longitudinal relational database.

#### Assessment of CoD from VA

For assessment of probable CoD from VA symptoms we used the computer-automated probabilistic model ‘InterVA-4 (version 4.02)’ in the structure of the 2012 WHO VA instruments (15). The computer model is relatively fast, low-cost, and produces consistent and comparable CoD in comparison with the physician's (either single or a panel) review of VA for allocating CoD (16–19). It speeds up VA interviews by eliminating the need for transcribing lengthy narratives related to death history (19, 20). It processes a range of items of information about the background characteristics and circumstances of a death, details of any illness (signs and symptoms), or conditions leading to death, and previous medical history in a mathematical model based on Bayes’ theorem, and produced likely CoD (21).

### Data analysis

AMK HDSS contributed to the INDEPTH multisite dataset, providing VA data and person-years of observations during 2004–2010 (20). HDSS data were used to compute percentage and rate (per 1,000 person-years) of deaths by age and sex for each site. VA data on symptoms and signs of illness or conditions collected were converted to the WHO 2012 standard (21). It may be noted that for 17 deaths no symptom or sign of illness or condition was recorded in VA and these deaths were excluded from the analysis of CoD. We ran InterVA-4 (version 4.02) with options of low prevalence of HIV/AIDS and malaria deaths in the surveillance sites to produce likely biomedical CoDs. The InterVA-4 yields, for each case, up to three possible causes with estimated probabilities or an indeterminate result. The estimated probabilities for the first, second, and third most likely CoD were all summed, and if the sum of their probabilities was less than 100%, the residual component was then assigned as being indeterminate. CoD were broadly grouped into communicable diseases (CDs), non-communicable diseases (NCDs), perinatal and neonatal causes, pregnancy-related deaths, injury and other external causes, or indeterminate. CSMFs per 100 deaths by sex within the sites and by sites were estimated to exhibit sex and areal differentials. The differentials were tested for statistical significance using *Z*-value at *p*<0.05.

## Results

The midyear population data in 2010 revealed that Abhoynagar had a smaller proportion of young population (age below 15) and larger proportion of working-age male population (aged 15–49) as compared to Mirsarai (Table 1). The proportion of old adults (aged 50–64) and elderly were comparable between the sites. The age-specific sex ratios of the male per 100 female person-years were less skewed in Abhoynagar than Mirsarai, with sex ratio being the lowest in the age group 15–49 due to higher out-migration of men aged 15–49 in the latter site. The average ages of the males and females (29.8 and 29.2, respectively) in Abhoynagar were higher than the average ages of the males and females (27.3 and 28.3, respectively) in Mirsarai.

**Table 1:** Age distribution (in%) and sex ratio of males per 100 females of the midyear population in 2010 in each site, AMK HDSS 2010

	Abhoynagar			Mirsarai		
		
Age group (in years)	Male (*n*=17,489)	Female (*n*=17,375)	Sex ratio (100*M/F)	Male (*n*=18,321)	Female (*n*=20,929)	Sex ratio (100*M/F)
0–4	8.7	8.6	100.9	10.9	9.1	105.5
5–14	19.6	18.8	104.9	22.8	19.8	100.9
15–49	53.2	55.6	96.3	48.6	54.9	77.5
50–64	13.0	11.3	115.8	12.2	10.7	99.4
65 +	5.5	5.6	98.9	5.5	5.6	86.6
Mean age±SD^a^	29.8±20.0	29.2±19.6	100.7	27.3±20.4	28.3±19.7	87.5

a Means and SDs (standard deviations) were estimated from individual's age.

*n*=size of the midyear population.

There were 1,384 deaths in Abhoynagar surveillance site and 1,847 deaths in Mirsarai surveillance site during 2004–2010, yielding crude death rates of 5.8 and 6.7 per 1,000 person-years, respectively (Table 2). Annual crude death rates and death rates in all age groups, except in the age group 1–14 did not exhibit declining trends in both sites during 2004–2010 (data not shown). There was a difference in age and sex patterns of mortality in these two rural sites. Infant, particularly neonatal, mortality rate (*p*<0.01) was critically higher in Abhoynagar than in Mirsarai, where mortality rates were higher in all age groups except in the infancy. The overall sex ratio of the male to female death rates was less skewed in Abhoynagar than Mirsarai, with a marked difference between age groups in each site. The sex ratios were more skewed in the neonatal, post-neonatal, and 1–4-year age groups in Abhoynagar than Mirsarai, where sex ratios were skewed in all age groups 5 years and above.

**Table 2:** Distribution of deaths, death rates,^a^ and sex ratios^b^ of death rates by age in Abhoynagar and Mirsarai sites, AMK HDSS 2004–2010

	Abhoynagar			Mirsarai			
		
Age group	# Deaths (%)	Death rate^a^	Sex ratio^b^	% (# Deaths)	Death rate^a^	Sex ratio^b^	*P*-value for the comparison of the death rates between sites
0–28 days^c^	156 (11.3)	34.3	174.0	131 (7.1)	21.8	108.6	*p*<0.01
29 days–11 months^c^	49 (3.5)	10.8	48.6	57 (3.1)	9.5	74.0	*p*<0.32
1–4 years	32 (2.3)	1.7	160.3	78 (4.2)	3.2	92.3	*p*<0.01
5–14 years	21 (1.5)	0.4	87.2	45 (2.4)	0.8	146.4	*p*<0.05
15–49 years	195 (14.1)	1.5	107.0	282 (15.3)	2.0	157.9	*p*<0.01
50–64years	199 (14.4)	7.8	118.6	327 (17.7)	11.8	177.1	*p*<0.01
65+years	732 (52.9)	59.4	107.6	927 (50.2)	63.5	128.8	*p*<0.15
All ages	1,384 (100.0)	5.8	114.0	1,847 (100.0)	6.7	135.2	*p*<0.01
#Person-years	237,876			275,853			

a Per 1,000 person-years of observation

b ratio of the male to female death rates (100*M/F)

c per 1,000 person-years in infancy.

### Differences in CoD in two surveillance sites

The distribution of CSMFs by site shows the differences in broad CoD categories (Table 3). It may be noted that the InterVA-4 could not assign a specific cause to 10.3% (10.9% in Abhoynagar and 9.8% in Mirsarai) of all deaths. CSMF due to CDs was lower (16.7% vs. 21.2%) in Abhoynagar than Mirsarai. The most common CDs were acute respiratory infections (ARI) including pneumonia and pulmonary tuberculosis with differences between the sites. CSMFs for ARI/pneumonia (9.4% vs. 7.1%) and tuberculosis (9.0% vs. 6.4%) were higher in Mirsarai than Abhoynagar. More than half of the deaths were caused by NCDs with no significant differences between the two sites (55.3 and 56.2% in Mirsarai and Abhoynagar, respectively). However, CSMF due to stroke was higher (19.3% vs. 16.5%) in Abhoynagar than Mirsarai, whereas CSMF due to cardiac diseases was higher in Mirsarai (11.6% vs. 8.9%). CSMF due to malignant neoplasms was 13.2% in each site. Malignancies were more frequent in the digestive system (5.4 and 5.6%, respectively) followed by the respiratory system (3.8 and 5.0%, respectively) in both sites. Maternal causes accounted for 0.8–0.9% of the deaths in each site, but perinatal and neonatal causes were higher (7.6% vs. 5.3%) in Abhoynagar than Mirsarai. Though CSMF due to injury and other external causes was comparable (7.5–7.7%) between the two sites, accidental drowning (2.3% vs. 1.4%) and road traffic accidents (1.9% vs. 1.0%) were more frequent in Mirsarai than Abhoynagar, whereas intentional self-harm was more frequent in Abhoynagar (3.5% vs. 1.5%).

**Table 3:** Cause-specific mortality fractions (in%) by sex and site, AMK HDSS 2004–2010

	Abhoynagar			Mirsarai		
		
Major causes of death	Male	Female	Total	Male	Female	Total
Communicable diseases	16.8	16.6	16.7	21.6	20.7	21.2**
ARI or pneumonia	5.5	8.8*	7.1	8.9	10.0	9.4*
Pulmonary tuberculosis	8.6	3.9**	6.4	10.4	7.3*	9.0**
Other infections	2.7	3.9	3.2	2.3	3.4	2.8
NCD	54.6	58.1	56.2	53.4	56.9	55.3
Stroke	17.8	20.9	19.3	13.8	20.0**	16.5*
Cardiac disease	10.2	7.3	8.9	13.5	9.2**	11.6
COPD or asthma	6.9	5.7	6.3	5.4	4.7	5.1
Neoplasm	12.6	13.8	13.2	13.9	12.3	13.2
Digestive system	4.1	7.5**	5.6	5.1	5.8	5.4
Respiratory system	5.5	1.8**	3.8	6.2	3.4**	5.0
Other neoplasm	3.0	4.6	3.7	2.6	3.0	2.8
Acute abdomen	1.4	2.2	1.8	2.0	4.4	3.1
Liver cirrhosis	1.1	1.5	1.3	1.2	0.7	1.0
Diabetes mellitus	3.1	3.9	3.5	2.9	2.7	2.8
Severe anaemia/malnutrition	0.5	1.1	0.8	0.5	1.2	0.8
Other NCD	1.0	1.6	1.2	0.7	1.7	1.2
Perinatal and neonatal causes	9.1	5.8*	7.6	5.1	5.6	5.3**
Neonatal pneumonia	3.5	2.6	3.1	1.9	1.5	1.7*
Birth asphyxia	1.4	1.4	1.4	1.7	1.3	1.5
Prematurity	1.4	0.2	0.9	0.5	1.1	0.7
Other neonatal cause	2.8	1.5	2.2	1.0	1.7	1.3
Maternal cause	NA	1.8	0.8	NA	2.0	0.9
Injury and other external causes	7.9	7.4	7.7	9.3	5.4	7.5
Accidental drowning	1.6	1.1	1.4	2.2	2.5	2.3
Road traffic accident	1.4	0.5	1.0	3.3	0.2**	1.9*
International self-harm	2.5	5.1**	3.5	1.3	1.7	1.5**
Assault	1.3	0.2*	0.8	1.2	0.4*	0.8
Other external cause	1.4	0.7	1.1	1.3	0.6	1.0
Indeterminate^a^	11.5	10.2	10.9	10.0	9.4	9.8
Number of deaths	743	641	1,384	1,016	831	1,847

ARI=acute respiratory infection; COPD=chronic obstetric pulmonary diseases; NA=not applicable; NCD=non-communicable diseases.

a Excluded 17 deaths for which no symptom or sign of illness was record in VA from determining cause of death, but included in the indeterminate.

* *p*<0.05

** *p*<0.01 (compared between ‘Male’ and ‘Female’ within the site or between totals of sites).

### Sex difference in CoD within the surveillance sites

The breakdown of CSMFs by sex shows biosocial differences in mortality risks (Table 3). Although the CSMF due to CDs exhibited sex parity in each site, the CSMF for pulmonary tuberculosis was higher for males than females in both sites (8.6% vs. 3.9% in Abhoynagar and 10.4% vs. 7.3% in Mirsarai) and for ARI/pneumonia it was higher for males in Abhoynagar (8.8% vs. 5.5%) only. CSMFs due to NCDs were comparable between females and males within the site. Stroke was more frequent (20.0% vs. 13.8%) among females in Mirsarai and cardiac disease was more frequent among males in both sites (13.5% vs. 9.2% and 10.2% vs. 7.3% in Abhoynagar). There was no sex difference in CSMF due to malignant neoplasms, but malignancies in the digestive system were more frequent among females (7.5% vs. 4.1%) in Abhoynagar, and in the respiratory system were more frequent among males in both sites (5.5% vs. 1.8% in Abhoynagar and 6.2% vs. 3.4% in Mirsarai). No sex difference was noted in CSMF due to diabetes mellitus and liver cirrhosis, but acute abdomen was more frequent among females than males (3.4% vs. 1.8%) in both sites.

Perinatal and neonatal causes exhibited sex differences in favour of females in Abhoynagar, and conditions relating to pregnancy accounted for 13.3% of the deaths of adult (aged 15–49) females. CSMF due to external causes exhibited no sex difference in Abhoynagar, but against males (9.3% vs. 5.4%) in Mirsarai. Although the frequency of accidental drowning did not vary by sex, road traffic accident (1.4% vs. 0.5% in Abhoynagar and 3.3% vs. 0.2% in Mirsarai) and assault (1.3% vs. 0.2% in Abhoynagar and 1.2% vs. 0.4% in Mirsarai) were more frequent among males than females, among whom intentional self-harm was more frequent, particularly in Abhoynagar (5.1% vs. 2.5%).

### The rank-order of CoD by age

Table 4 shows marked variations in the rank-order (measured with CSMFs) of top 10 CoDs between age groups. It may be noted that the percentage of indeterminate cases varied by age; highest (27.3%) among the neonates, and lowest (1.7%) among children aged 1–4. Neonatal deaths were due mostly to ARI/pneumonia (25.7%), followed by birth asphyxia (16.8%), prematurity (9.0%), and sepsis (4.1%), summing up to 55.6% of the deaths. ARI/pneumonia (65.4%) and diarrhoea (14.4%), accounted for 79.8% of the post-neonatal deaths. More than half (55.4%; with accidental drowning accounting for 49%) of the child (aged 1–4) deaths was due to injury and other external cause, followed by ARI/pneumonia (26.3%), diarrhoea (9.2%), and malnutrition (4.2%), totalling to 95.1% of the deaths. The leading CoD of older children (aged 5–14) were injury (38.7%, drowning accounting for 9.8%), ARI/pneumonia (13.6%), tuberculosis (7%), and acute abdomen (6.9%), totalling to 66.2% of the deaths.

**Table 4:** Cause-specific mortality fractions [in%] by age group of the deceased (both sites combined), AMK HDSS 2004–2010

0–28 days (# deaths=287)	29 days–<1 year (# deaths=106)	1–4 years (# deaths=110)	5–14 years (# deaths=66)	15–49 years (# deaths=477)	50–64 years (# deaths=526)	65+ years (# deaths=1,659)
ARI/pneumonia [25.7%]	ARI/pneumonia [65.4%]	Injury [55.4%] Drowning [49.0%]	Injury [38.7%] Drowning [9.8%]	Injury [23.5%] Self-harm [11.9%]	Neoplasm [19.7%]	Stroke [26.8%]
Birth asphyxia [16.8%]	Diarrhoea [14.4%]	ARI/pneumonia [26.3%]	ARI/pneumonia [13.6%]	Neoplasm [16.5%]	Stroke [17.9%]	Neoplasm [14.6%]
Prematurity [9.0%]	Meningitis/encephalitis [2.6%]	Diarrhoea [9.2%]	Tuberculosis [7.0%]	Cardiac diseases [11.8%]	Cardiac diseases [17.4%]	Cardiac diseases [11.3%]
Sepsis [4.1%],	Injury [2.2%]	Malnutrition [4.2%]	Acute abdomen [6.9%]	Tuberculosis [9.8%]	Tuberculosis [10.5%]	Tuberculosis [8.8%]
Malformation [2.4%]	Malnutrition [1.8%]	Tuberculosis [1.9%]	Meningitis/encephalitis [3.1%]	Stroke [6.4%]	COPD/asthma [5.2%]	COPD/asthma [8.7%]
Meningitis/encephalitis [2.3%]			Stroke [3.0%]	Maternal cause [6.0%]	Acute abdomen [3.8%]	ARI/pneumonia [6.9%]
			Cardiac disease [2.7%]	ARI/pneumonia [4.1%]	Injury [3.8%]	Diabetes [4.4%]
				Acute abdomen [3.3%]	Diabetes [3.6%]	
Other [12.3%]	Other [5.5%]	Other [3.2%]	Other [13.4%]	Other [10.2%]	Other [10.5%]	Other [9.2%]
Indeterminate [27.3%]	Indeterminate [7.1%]	Indeterminate [1.7%]	Indeterminate [11.6%]	Indeterminate [8.4%]	Indeterminate [7.7%]	Indeterminate [9.3%]

The most common CoD for young adults (aged 15–49) was injury (23.5%, self-harm accounting for 11.9%), followed by malignant neoplasm (16.5%), cardiac diseases (11.8%), tuberculosis (9.8%), stroke (6.4%), and ARI/pneumonia (4.1%). These accounted for 72.1% of the deaths. Deaths from conditions related to pregnancy accounted for 13.3% of the adult female deaths. Of the older adult (aged 50–64) deaths, malignant neoplasms (19.7%), stroke (17.9%), cardiac disease (17.4%), tuberculosis (10.5%), and COPD including asthma (5.2%) accounted for 70.7% of the deaths. The leading cause of elderly (aged 65 and older) deaths was stroke (26.8%), followed by malignant neoplasm (14.6%), cardiac diseases (11.3%), tuberculosis (8.8%), COPD (8.7%), ARI/pneumonia (6.9%), and diabetes (4.4%), totalling to 81.5% of all the deaths.

## Discussion

The results revealed critically higher mortality, particularly among male neonates in Abhoynagar than Mirsarai, which does not match with lower crude death rate and use of maternal health services. In 2009, in Abhoynagar, 31% of the mothers who gave live births received recommended 4+ antenatal care visits and 64% received 1+ postnatal care visit compared to 24 and 16%, respectively, in Mirsarai (14). However, the rates of institutional deliveries of live births in 2009 were comparable between these two sites (21% in Abhoynagar and 23% in Mirsarai). Male neonates are biologically more vulnerable, but vulnerability was critically higher in Abhoynagar, which is a surprise and we do not have any reasonable explanation. However, high neonatal mortality rate could be due to high rates of teenage marriage and fertility and colder ambient temperature in the winter season in Abhoynagar compared to Mirsarai (14, 22). Teenage motherhood is associated with increased risks for pre-term delivery, low birth weight, and neonatal mortality (23). Moreover, perinatal mortality steeply increased with a decrease in temperature in the winter below the temperature of 23°C (24).

The mortality rates in all age groups except in infancy were lower in Abhoynagar than Mirsarai. This difference was due mostly to less frequent deaths from CDs, particularly from ARI/pneumonia and tuberculosis in Abhoynagar. Such site-specific statistics are important because they could help health managers in local-level planning of health services and designing appropriate measures that will save lives and improve economic conditions.

Sex differences in mortality rates in the age groups 5 years and above were in favour of females and they were much higher in Mirsarai than Abhoynagar. Why males in Mirsarai experienced excess mortality compared to females of the same age groups may be explained by the lower population sex ratio, which is determined by more out-migration (national or international) of males. The overall population sex ratio of males to 100 females in 2010 was 87 in Mirsarai compared to 101 in Abhoynagar, although the sex ratios in the age group 0–4 were comparable (101 vs. 105) between sites (Table 1). The sex ratio in the age group 15–49 was even lower (77 vs. 96) in Mirsarai, perhaps due to economic and labour migration. In general, healthy individuals are more likely to undertake migration, leaving the less healthy ones at home. The healthy migrant effect in terms of mortality was observed in Germany, comparing Turkish migrants to Germans locals, and it could be due to self-selection at the time of immigration (25).

The CoD patterns revealed sex differences in the health burdens of specific NCDs, tuberculosis, and injuries. Parallel sex differentials were observed in the distribution of certain morbidity conditions in the Bangladesh Demographic and Health Survey (BDHS) 2011, which screened for prevalence of hypertension and diabetes in women and men aged 35 and older (2). More women than men (32% vs. 19%) were hypertensive, but diabetes (11%) was similar in both sexes. More than half of them, however, were not aware that they had the diseases. Another study noted similar sex differentials in the risk factors of NCDs. More men aged 25–64 used tobacco products (68.2% vs. 32.7%) than women of similar ages. Women were more often overweight (15.2% vs. 10.8%) compared to men (26).

As expected and also noted with the physician-coded CoD in a large-scale national survey BDHS 2011 (2), the rank-order of the InterVA-4-coded CoD changed markedly by age group despite the methodological differences (in terms of questionnaires used, length of the recall period, assessment and categorisation of CoD, and time period). Except for neonates, the rank-order of the physician-coded CoD of the post-neonates and children (aged 1–4) is comparable with the rank-order of the InterVA-4 coded CoD. There is a large variation in the distribution of CoD of neonates assessed by the InterVA-4 and by the physician. The leading physician-coded CoD was ‘possible serious infections’ (24.3%), for which there is no comparable category in the InterVA-4. However, the sum of the physician-coded deaths due to infections (i.e. possible serious infections, ARI/pneumonia, and diarrhoea) was 34.6%, which is comparable to the sum of the InterVA-4-coded deaths due to ARI/pneumonia, sepsis, meningitis, and diarrhoea, totalling to 32.5%.

The rank-order of the top two causes of post-neonatal deaths coded by physician and InterVA-4 are comparable; ARI/pneumonia (66% in InterVA-4 and 52.9% in BDHS 2011), followed by diarrhoea (14.6% in InterVA-4 and 7.5% in BDHS 2011) (2). The InterVA-4 coded causes of child deaths are also comparable in rank-order with the physician-coded causes; drowning (49.0% vs. 42.6%), followed by ARI/pneumonia (26.3% vs. 21.7%). Also the physician-coded top five CoDs of females aged 15–49 reported in the Bangladesh Maternal Mortality and Health Care Survey 2010 were found comparable with the InterVA-4 coded top five CoDs of females of the same age group in these two sites. The comparability of the InterVa-4 coded CoDs, despite several methodological differences, with the physician-coded CoDs reveals the potentials of VA in HDSS sites to be used for planning and monitoring of the disease burdens not only in these sites but also in the regions as well as in the country with obvious subtle differences.

The patterns of CoDs revealed the prominence of NCDs compared to CDs in both sites, which has implications for the public health system to respond. Management of most NCDs is available at tertiary level hospitals, but it is not for reduction and prevention of the risks of developing NCDs. Many NCDs are, however, amenable to prevention through behavioural changes. Lifestyle and behaviours are linked to 20–25% of the global burden of disease, which is likely to increase rapidly in poorer countries in the process of rapid urbanisation and demographic transition (27). In Bangladesh, consumption of vegetables and fruits and regular exercise are at a low level, whereas use of tobacco products, excessive intake of salt, and abuse of substances are considerably high (26). These risk factors are shared by a number of NCDs, so health-promotion directed towards these risk factors will address most simultaneously (28). Strengthening behaviour change activities at the community level for promoting risk-reducing behaviour, expanding screening facilities for early detection of NCDs, and increasing compliance with effective medication can lower the disease burden, health expenditure, and loss of productivity and national health expenditure.

Injury and other external causes are the leading cause of mortality in the age groups 1–4, 5–14, and 15–49 in rural communities. Particularly accidental drowning accounted for 88% of the injury-related deaths in the 1–4 age group and 25% in the 5–14 age group. Evidence-based interventions and community awareness are needed for lowering such deaths. One-fourth (24%) of the adult deaths were due to injury and other external causes, half (51%) of them were due to self-harm, and another 14% were due to assault. Physical and mental assaults often provoke self-harm, thus may have underestimated the share of assault. Violence against young women is quite high in South Asia including Bangladesh and is often perpetrated by their husbands or his family members (29–31). Assessment of CoD from VA provided usually by victim's family members cannot divulge true cause – a limitation of VA collecting from the deceased's family members.

Evidence-based planning of health services and logistics requires reliable and up-to-date public health statistics including CoD. In Bangladesh, the civil registration, particularly deaths with medical certification is too incomplete to generate such statistics. The national SVRS administered by the Bangladesh Bureau of Statistics, the Government of Bangladesh, records vital events including deaths from a representative sample of the population. Introduction of VA into the national SVRS and computer-automated coding of VAs of a nationally representative sample of deaths can generate CoD statistics on a regular basis for use in public health planning until the civil registration system well functions.

Health burdens posed by CDs, NCDs, and external causes are the major challenges to improving population health. The government Health, Population, and Nutrition Sector Development Program for 2011–2016 includes a plan for expanding access to health services for controlling conventional (hypertension, diabetes, cancer, COPD, psychiatric illness, etc.) and non-conventional (road safety and injury and violence against women) NCDs (32). The operation plan includes conducting training on NCD screening and management for health care providers at district and subdistrict levels, organising awareness-building workshops on injuries, and pilot screening and management of selected NCDs at the subdistrict level facilities, gradually expanding to the lower level facilities. The private health sector, particularly pharmacies in urban and rural areas, and workplace based prevention and screening, can play an important role in screening and referral. Prevention, early detection, and compliance with effective medication can save national health expenditure as NCDs require long-term care and bring catastrophic economic consequences for high out-of-pocket payments. Appropriate measures to minimise the catastrophic effects may include, but are not limited to, community-based health insurance, credit to cushion income loss, and social safety net programmes.

## Conclusions

In conclusion, analyses of VA symptom data using InterVA-4 model revealed health burdens, with leading causes being stroke, neoplasms, cardiac diseases, ARI/pneumonia, tuberculosis, and COPD. External causes were more frequent among males, but self-harm was higher in Abhoynagar, particularly for females. The primary health care centres, currently equipped to manage CDs which is the outmost concern, must be equipped for prevention, screening, and management NCDs as well.

## References

[1] BDHS (2004). Cause of death in children under-five years of age. Bangladesh demographic and health survey 2004.

[2] BDHS (2011). Cause of death in children under age 5. Bangladesh demographic and health survey 2011.

[3] BMMS (2001). Adult female mortality: levels and causes. Bangladesh maternal health services and maternal mortality survey 2001.

[4] BMMS (2010). Adult female mortality: levels and causes. Bangladesh maternal mortality and health care survey 2010.

[5] Bangladesh Bureau of Statistics (2011). Population and housing census 2011: preliminary results.

[6] Alam N, Chowdhury HR, Bhuiyan MA, Streatfield PK (2010). Causes of death of adults and elderly and healthcare-seeking before death in rural Bangladesh. J Health Popul Nutr.

[7] Bangladesh Bureau of Statistics (2012). Report on sample vital registration system.

[8] Management Information System (2012). Health bulletin 2012. http://dghs.gov.bd/bn/licts_file/images/Health_Bulletin/HealthBulletin2012_en.php.

[9] World Health Organization (2005). WHO technical consultation on verbal autopsy tools.

[10] Baiden F, Bawah A, Biai S, Binka F, Boerma T, Byass P (2007). Setting international standards for verbal autopsy. Bull World Health Organ.

[11] Fottrell E, Byass P (2010). Verbal autopsy: methods in transition. Epidemiol Rev.

[12] Soleman N, Chandramohan D, Shibuya K (2006). Verbal autopsy: current practices and challenges. Bull World Health Organ.

[13] Banglapedia (2012). Climatic zone. http://www.bpedia.org/C_0289.php.

[14] Lindeboom W, Das SC, Ashraf A (2011). Health and demographic surveillance report 2009- Abhoynagar and Mirsarai.

[15] Byass P, Chandramohan D, Clark SJ, D'Ambruoso L, Fottrell E, Graham WJ (2012). Strengthening standardised interpretation of verbal autopsy data: the new InterVA-4 tool. Glob Health Action.

[16] Byass P, Fottrell E, Dao LH, Berhane Y, Corrah T, Kahn K (2006). Refining a probabilistic model for interpreting verbal autopsy data. Scand J Public Health.

[17] Murray CJ, Lozano R, Flaxman AD, Vahdatpour A, Lopez AD (2011). Robust metrics for assessing the performance of different verbal autopsy cause assignment methods in validation studies. Popul Health Metr.

[18] Byass P (2007). Who needs cause-of-death data?. PLoS Med.

[19] King G, Lu Y (2008). Verbal autopsy methods with multiple causes of death. Statist Sci.

[20] INDEPTH Network (2014). INDEPTH Network Cause-Specific Mortality – Release 2014. http://www.indepth-network.org.

[21] Leitao J, Chandramohan D, Byass P, Jakob R, Bundhamcharoen K, Choprapawon C (2013). Revising the WHO verbal autopsy instrument to facilitate routine cause-of-death monitoring. Glob Health Action.

[22] Alam N, Lindeboom W, Begum D, Streatfield PK (2012). The association of weather and mortality in Bangladesh from 1983–2009. Glob Health Action.

[23] 
Xi-Kuan C, Wen SW, Fleming N, Demissie K, Rhoads GG, Walker M (2007). Teenage pregnancy and adverse birth outcomes: a large population based retrospective cohort study. Int J Epidemiol.

[24] Hashizume M, Wagatsuma Y, Hayashi T, Saha SK, Streatfield K, Yunus M (2009). The effect of temperature on mortality in rural Bangladesh – a population-based time-series study. Int J Epidemiol.

[25] Razum O, Zeeb H, Rohrmann S (2000). The ‘healthy migrant effect’ – not merely a fallacy of inaccurate denominator figures. Int J Epidemiol.

[26] Ahmed SM, Hadi A, Razzaque A, Ashraf A, Juvekar S, Ng N (2009). Clustering of chronic non-communicable disease risk factors among selected Asian populations: levels and determinants. Glob Health Action.

[27] World Health Organization (2008). 2008–2013 action plan for the global strategy for the prevention and control of non-communicable diseases. http://whqlibdoc.who.int/publications/2009/9789241597418_eng.pdf.

[28] Lawes CM, Vander HS, Rodgers A (2008). Global burden of blood-pressure-related disease, 2001. Lancet.

[29] Bates LM, Schuler SR, Islam F, Islam K (2004). Socioeconomic factors and processes associated with domestic violence in rural Bangladesh. Int Fam Plan Perspect.

[30] Koenig MA, Ahmed S, Hossain MB, Khorshed Alam Mozumder MAB (2003). Women's status and domestic violence in rural Bangladesh: individual- and community-level effects. Demography.

[31] World Health Organization (2003). WHO 2003 – multi-country study on women's health and domestic violence against women.

[32] Ministry of Health and Family Welfare (MOHFW), Government of the People's Republic of Bangladesh (2011). Health, Population and Nutrition Sector Development Program (HPNSDP) (July 2011–June 2016). Volume 1, Program Implementation Plan (PIP).

